# Clinical evaluation and management of a 45-year-old man with confusion, psychosis, agitation, stereotyped behavior, and impaired speech

**DOI:** 10.1155/2022/8162871

**Published:** 2022-05-17

**Authors:** Xiaolin Deng, Paulo J. Negro, Patrick L. Jung, Christopher M. Marano, Stephanie Knight, Seshagiri R. Doddi, Nana Y. A. Nimo, Rachel M. LeMalefant, Drew A. Myers, Andrea K. Haake, Rebecca Chandler

**Affiliations:** Department of Psychiatry, University of Maryland School of Medicine, 22 S Greene Street, Baltimore MD 21201, USA

## Abstract

Our patient Mr. A is a mentally and physically disabled gentleman. He was first diagnosed with bipolar disorder as a teenager. He incurred a lumbar spinal injury due to a motor vehicle incident in his 20s which led to weakness, numbness, and frequent infection over both of his lower extremities. He also developed alcohol addiction over the course of his life. Mr. A presented to our facility with complicated neuropsychiatric symptoms. By adopting various clinical strategies, we were able to control his symptoms of agitation, self-harm, mood swings, and stereotyped behavior. However, we were not able to improve his neurocognitive functioning or speech impairment which seemed to become severe and irreversible in a period of a few months. We felt disappointed and perplexed by the mixed treatment responses. To understand Mr. A's clinical presentation, various laboratory tests and imaging studies were performed. Different psychotropic medications were used to manage his symptoms. Gradually, we felt that we were able to understand this case better clinically and etiologically. His bipolar disorder, alcohol addiction, and physical injury had likely all contributed to his neuropsychiatric symptoms, directly or indirectly. It is highly possible that an alcohol-related progressive dementia along with his chronic bipolar disorder played a key role in the progression of his brain neurodegeneration. Also, Wernicke-Korsakoff syndrome could reasonably be considered having developed during his clinical course. Moreover, the fluctuation of the patient's neuropsychiatric symptoms we observed during his hospitalization reflects the increased vulnerability of the human brain under sustained neurodegeneration.

## 1. Introduction

Psychiatric medicine has made significant progress in managing complicated psychotic and behavioral symptoms since chlorpromazine was first effectively used to treat psychotic symptoms in the early 1950s [1]. Psychiatrists have also accumulated more scientific knowledge to associate patients' clinical symptoms with dysfunctional brain systems. However, managing brain neurodegeneration and its related psychotic and behavioral symptoms remain a challenge for psychiatrists today.

Investigation into brain neurodegeneration has attracted the attention of many basic and clinical scientists. It is known that brain neurodegeneration can occur rapidly or gradually [2–5]. Clinically, it is highly important for psychiatrists to assess patients' cognition and intervene on reversible causes of brain neurodegeneration. It has been well documented that chronic alcohol use can cause brain neurodegeneration due to alcohol neurotoxicity [2, 3]. In addition, alcohol use can also lead to Wernicke's encephalopathy and Korsakoff syndrome. The clinical presentation of each of these two conditions can be diverse and complicated [6–9]. Moreover, patients with severe mental illnesses such as schizophrenia and bipolar disorder can also gradually develop cognitive deficits [4, 10].

Clinically, it may be easy to recognize a patient's cognitive deficits; however, understanding the underlying etiology is often more difficult. Patients with chronic mental illnesses often suffer from comorbidities of addiction, physical disability, and malnutrition which can also contribute to their physical and mental decline. As their clinical pictures become more complicated, treatment can be very challenging.

## 2. Case Presentation

Mr. A, a 45-year-old single Caucasian male, was brought to a local emergency department (ED) by his friend due to worsening foot pain on June 7^th^, 2021. His foot pain had been a chronic issue for him. He had previously fractured his lumbar spine around 2000, which led to lower extremity weakness and numbness. Because of this weakness, he was dependent on a cane and his feet had gradually become deformed. He ended up having tendon and bony destruction in both ankles, which led him to wear braces on both ankles. Over time, his use of the braces led to the development of repetitive abscesses in his ankles.

On the way to the ED, Mr. A was his normal self, “joking and laughing” according to his friend. At the ED, Mr. A was found to have right ankle erythema and mild elevation in his white count. Foot computerized tomography (CT) showed cellulitis and abscess on the lateral right ankle with destruction of cartilage, widening of the joint space, and multiple bony fragments. He was admitted to the hospital for the treatment of his right ankle cellulitis. He completed a full course of Augmentin antibiotic treatment while in the hospital.

During this hospital stay, Mr. A was noted to develop symptoms of depression, paranoia, and intermittent agitation. Psychiatry was consulted. Mr. A reported to the psychiatrist and staff that he was hearing voices and felt depressed. He also had episodes of uncontrollable crying which were noted by the psychiatrist. Occasionally, he seemed to be confused, but it was reported that he was able to engage in meaningful conversation and answer most questions appropriately. Preliminary psychiatric diagnoses during this hospitalization were bipolar disorder and delirium. A few medications were ordered by the psychiatrist to address Mr. A's symptoms. After completing the course of antibiotics for his right ankle cellulitis, Mr. A was discharged to a local nursing home on June 24^th^, 2021. Psychotropic medications at discharge included escitalopram 20 mg daily, mirtazapine 7.5 mg at bedtime and risperidone 1 mg in the morning and 3 mg at bedtime.

On August 3^rd^, 2021, Mr. A was sent to a nearby ED from the nursing home following a suicide attempt by drowning himself in a toilet. He reported feeling depressed and suicidal. He was also experiencing auditory hallucinations, paranoia, and persecutory delusions. No visual hallucinations were reported. His laboratory workup at ED was largely unremarkable, except for a urinalysis which indicated a possible urinary tract infection (UTI). Antibiotics were started, and he was admitted to the medical floor for further management of his UTI. The next day, the consulting psychiatrist noticed that he seemed to be catatonic. Subsequently, he was transferred to the psychiatric ward in the same hospital with the diagnoses of bipolar I disorder, most recent episode mixed with psychotic features; metabolic encephalopathy secondary to UTI and dehydration; and catatonia. Lorazepam was started to treat his catatonia which seemed to be effective. However, Mr. A seemed to be getting more and more psychotic and agitated. His speech was noticed to be repetitive, meaningless, and unintelligible. He was frequently hitting his head with his fists. Soft mitts were placed on his hands. The local psychiatrist then contacted the University of Maryland Medical Center (UMMC) Psychiatry Department. Subsequently, Mr. A was transferred to UMMC for further investigation and treatment including possible electroconvulsive therapy (ECT).

On August 28^th^, 2021, Mr. A arrived at UMMC's adult psychiatric unit. His transportation was not as smooth as expected. On his way to UMMC, he was restrained with three wrap belts. Bilateral mittens were also used as he was frequently hitting himself on the face. During his admission, he was kicking both of his legs up in the air and banging his head and upper body onto the stretcher in a rhythmic fashion. His speech was largely unintelligible except stating, “God bless you” repeatedly, as well as repeating phrases that were said by staff. Upon being released from the restraints, Mr. A continued to be restless, screaming, hitting himself on the face, slamming his upper body, and kicking. He required a physical hold to apply 4-point restraints to prevent self-harm. His psychotropic medications at the time of transfer to UMMC included carbamazepine 200 mg twice daily, olanzapine 20 mg at bedtime, escitalopram 20 mg daily, and clonazepam 1 mg twice daily. Over the next few hours, olanzapine 10 mg intramuscular (IM) injection and clonazepam 1 mg per os (PO) were administered which did not diminish Mr. A's agitation. Benadryl 50 mg PO and lorazepam 2 mg PO were also given to him, and eventually, he calmed.

It was quickly noticed by the UMMC psychiatry team that Mr. A had demonstrated symptoms not only of psychosis and agitation but also of confusion, stereotyped behavior, and speech impairment. Confabulation was also suspected after collateral information from his friend had been obtained, as Mr. A was often mumbling about things that had not actually occurred in his life (e.g., having a wife and children). Given the relatively rapid onset of these behaviors, extensive studies were ordered to rule out underlying neurologic and autoimmune illnesses.

Neurology and Internal Medicine were consulted. A brain magnetic resonance imaging (MRI) study was obtained. Autoimmune markers were investigated. A lumbar puncture was performed, and cerebrospinal fluid (CSF) was tested. Despite these intensive studies, all test results came back negative, including a normal thiamine level. Despite this normal value, however, there was still a concern for Korsakoff syndrome given Mr. A's history of heavy alcohol drinking, poor nutrition (living on “junk food” for years), complicated symptoms, and no previous treatment with intravenous (IV) thiamine while at the outside hospitals. High-dose IV thiamine was subsequently administered for a few days. This was followed by thiamine PO indefinitely. Unfortunately, this supplementation was ineffective at reversing his current symptoms.

In the first weeks after his admission, management of Mr. A's agitation and self-harming behavior was the predominant, challenging task. Carbamazepine was initially increased to 200 mg three times daily, and olanzapine, Benadryl and hydroxyzine were frequently used as needed for agitation; but these agents failed to improve his agitation. Notably, lorazepam IM or PO administration seemed to be moderately effective in addressing his agitation. Therefore, olanzapine and carbamazepine were tapered off. Clonazepam was continued. Lorazepam 1-2 mg IM/PO were then used routinely to treat his agitation. Seventeen days after admission, clonazepam was switched to lorazepam and the total lorazepam dose was increased to 2 mg three times daily to deal with Mr. A's agitation. This strategy seemed to work well. In addition, clozapine was started to treat his psychotic symptoms including hallucinations, disorganized thinking, and echolalia.

With these medication changes, Mr. A's agitation and psychosis were largely controlled. He was able to participate in simple occupational therapy exercises. He did not demonstrate any positive psychotic symptoms. He was occasionally able to carry out short meaningful conversations. One day, he told his treatment team that he was previously on disability due to a back injury and “the way I walk.” He mentioned to the team that he had a past medical history of asthma and was initially diagnosed with bipolar disorder in his late teens and was treated with Lithium. He also stated that his first psychiatric hospitalization was in 1999 after his father committed suicide. He shared that he had been hospitalized a few times due to episodes of his bipolar disorder in the past. He had one previous suicide attempt by hanging himself. He acknowledged that he used to drink “a few beers” daily. His medical record indicated that he often consumed more than thirty cans of beer weekly. He denied using illicit drugs. Mr. A said that he had previously worked in construction but had been on disability since a motor vehicle accident in 2000. Since this accident, he required braces to walk. Although he was able to answer some of our questions casually from time to time, he was not able to concentrate on more formal tasks and conversation. Several attempts were made to assess his memory and cognitive functioning using the Montreal Cognitive Assessment (MoCA) and Mini-Mental State Examination (MMSE); this formal testing was not possible due to his poor concentration.

Although Mr. A's clinical improvement progressed favorably to certain degree, the treatment team remained perplexed by his significant cognitive impairment, stereotyped behavior, restlessness, and speech impairment. Since the diagnosis for Mr. A's illness was still unclear, obtaining more collateral information was a priority. Phone calls were made to his friend, as well as the psychiatrist who had evaluated and treated Mr. A in June of 2021. His friend reported that Mr. A seemed to be an “average normal guy.” He was not aware of Mr. A taking any medicine for at least fifteen years because “he did not like taking any pills.” Mr. A did not have any speech impairment or neurocognitive deficits that were noticed by his friend. However, Mr. A did drink alcohol regularly and “acted like an alcoholic”, getting drunk frequently. His friend shared that Mr. A had stopped drinking alcohol for two and half months prior to his hospitalization in June of 2021 because he was on antibiotics. The psychiatrist who evaluated Mr. A during the June 2021 hospitalization confirmed that he was able to carry on a fair conversation at that time. No clinical neurocognitive testing was performed at the outside hospital.

This collateral information suggested that Mr. A's neurocognitive decline, speech impairment, and stereotyped behavior developed recently while he had been recovering in the nursing home facility. With this timeline in mind, the treatment team considered a few possibilities to explain his symptoms including medication side effects, frontotemporal dementia, and conversion disorder with speech impairment. To determine whether side effects such as disinhibition and dyskinesia from his psychotropic medications were contributing to Mr. A's clinical presentation and stereotyped behaviors, lorazepam and clozapine doses were gradually decreased. Unfortunately, these changes almost immediately resulted in new and worsening symptoms. Mr. A became withdrawn and was demonstrating psychomotor retardation and catatonia. He would not eat or interact with staff. His memory loss and speech impairment had not improved as expected. To reverse these new symptoms, clozapine and lorazepam doses were gradually reintroduced over the course of a few days but titrated to lower levels than previously administered. Valproic acid was also started to replace some of the lorazepam dose.

With the reintroduction of medications, Mr. A was quickly getting back to his previous psychiatric baseline, thus making medication side effects an unlikely cause of his agitation. Because his speech deficits did not respond to medication adjustments, conversation disorder with speech impairment was thought to be highly unlikely. Geriatric psychiatrists were consulted for further collaboration. It was suggested that a positron emission tomography (PET) scan be completed; but the PET scan did not confirm any specific neurodegenerative disease including the findings typically seen in frontotemporal dementia. In addition, Mr. A was determined to be an inappropriate candidate for ECT as his behavioral and mood symptoms seemed to be under control. Thus, ECT was never pursued.

In addition to his psychiatric symptoms, Mr. A was bladder and bowel incontinent. During his hospitalization, urinalyses were tested multiple times during periods of increased agitation. He received antibiotics for UTIs in our facility. It was noticed that his agitation, restlessness, and mood swings would get worse before a UTI was diagnosed and then get better following treatment of the UTI.

As Mr. A's hospitalization continued, some degree of stability had been achieved using a combination of medications which included clozapine, lorazepam, valproic acid, and galantamine. The treatment team believed that Mr. A had reached a new baseline of mental and physical functioning. He was no longer agitated or engaged in self-injurious behaviors. He was able to participate in occupational therapy and nursing assessments. He could answer simple questions using single words or short phrases, often with interjections of verbigerated nonsensical speech. This baseline functioning was dramatically altered after the patient tested positive on a routine screening for COVID-19 on January 6^th^, 2022.

Mr. A was initially asymptomatic and without derangements in his vital signs. On January 9^th^, 2022, it was noticed by nursing staff that the patient developed a slight cough, congestion, and increased nonpurposeful movements. He was observed to be mumbling constantly to himself, yelling out, flailing his arms, attempting to strike staff during blood draws, and hitting his hand repeatedly on the side of his bed. Notably, these were some of the same behaviors observed early in his admission to UMMC. On the morning of January 10^th^, 2022, Mr. A's oxygen saturation was recorded to be fluctuating between 92 and 94%. Internal Medicine was consulted, and chest X-ray imaging was obtained which showed opacities in the mid and lower lung zones bilaterally, perhaps due to multifocal pneumonia. Laboratory testing revealed that Mr. A's d-dimer was within normal limits, diminishing the need for computed tomography angiography (CTA) imaging to rule out a pulmonary embolism. Mr. A never needed Remdesivir or Decadron for his COVID-19 infection, as his oxygen saturations were maintained above 94% on room air after January 10^th^, 2022. His COVID-19 symptoms were treated symptomatically, and Lorazepam 1-2 mg PO was utilized a few times for his increased agitation. Gradually, Mr. A returned to his baseline functioning in a few days and was considered recovered from his COVID-19 infection on January 17^th^, 2022. Mr. A later received a COVID-19 booster shot after he recovered, as he had previously received two doses of Pfizer's COVID-19 vaccine in May of 2021.

## 3. Discussion

Mr. A's unique and abrupt clinical findings led to a broad differential. As such, an extensive workup was performed to figure out the etiology of Mr. A's symptoms. A CT of his head was previously completed at the outside hospital and the result was unremarkable. Neurology and Internal Medicine were involved at UMMC and suggested specific studies. Firstly, to rule out the possible neurologic illnesses, electroencephalogram (EEG), MRI, and CSF studies were completed. These tests were largely unremarkable. Secondly, to rule out the possibility of underlying autoimmune illnesses, specific test panels were ordered. The results of these tests were mostly within normal limits or did not warrant further investigation. Genetic testing was not performed because there were no findings and clinical signs which raised concern for a possible genetic illness.

After completion of these extensive studies, the explanation for Mr. A's complicated symptoms seemed to rest with psychiatry. His history of heavy alcohol drinking, chronic bipolar disorder, and poor self-care for many years were all considered. It is suspected that Mr. A had developed brain neurodegeneration due to multiple etiologies, including progressive alcohol-induced dementia, ongoing bipolar disorder episodes, repeated infections, and metabolic encephalopathy.

Wernicke-Korsakoff syndrome can also be considered a culprit for Mr. A's suspected brain neurodegeneration. The variables which support this diagnosis in Mr. A's case include the following: (1) Mr. A did have a long history of heavy alcohol drinking. (2) Mr. A had frequent foot infections and had several surgeries for his back and feet. Frequent infections and surgeries may contribute to thiamine deficiency and subsequently cause Wernicke-Korsakoff syndrome [5–7]. (3) Mr. A's body mass index was 19.5 which was within normal range, but he was dependent on “junk food” for years due to his difficulty ambulating and poor self-care. Malnutrition was reasonably suspected in Mr. A's case and can be an exacerbating factor for Wernicke-Korsakoff syndrome, as malnutrition alone can cause Wernicke-Korsakoff syndrome [5, 7, 11]. (4) Mr. A had not received any IV thiamine or high dose PO thiamine prior to his admission to UMMC. He had been given a daily multivitamin at the previous facility, but the thiamine dose contained in this supplement has generally been considered ineffective in treating Wernicke-Korsakoff syndrome in patients with a long history of heavy alcohol drinking [10–12]. (5) Mr. A may have developed Wernicke's syndrome previously, without it being recognized or treatment of it considered, as he was having episodes of “delirium” in the outside hospital in June of 2021. It has been documented that most patients with Korsakoff syndrome developed the condition following inadequately treated or misdiagnosed Wernicke encephalopathy [12, 13]. Unfortunately, no formal ophthalmological examination was conducted for Mr. A during his hospitalizations. No abnormal eye movements such as ophthalmoplegia, nystagmus, anisocoria, or outward gaze palsy were documented. (6) Although Wernicke encephalopathy can be considered a progressive disease pathologically [14], Korsakoff syndrome is largely considered to be irreversible clinically [12, 15, 16]. It has been reported that neurons could be irreversibly damaged by a lack of thiamine, thus failing to respond to thiamine treatment even when presented with adequate amounts [9, 17]. Bolus IV thiamine infusion is mostly considered unhelpful after patients have passed the acute stage of Wernicke syndrome [6, 12]. This could explain Mr. A's failure to improve following his IV thiamine infusion. Also, Korsakoff syndrome may not be purely caused by thiamine deficiency but also due to deficiencies in other nutrients or via alcohol-induced neurotoxicity [8, 12, 18]. (7) Neuronal degeneration in the mammillary bodies, the characteristic neuropathology seen in patients with Wernicke-Korsakoff syndrome, was not detected on Mr. A's brain MRI. However, his MRI did show some nonspecific brain abnormalities in the periventricular white matter of both cerebral hemispheres. The periventricular area is another brain region reportedly affected in Korsakoff syndrome [9, 13, 14, 19].

In summary, Mr. A's clinical presentation and course highlights a complicated and unique case. From this and other reported cases, we may emphasize that patients with chronic mental illnesses are more likely to have comorbid addiction and physical illnesses. These patients are particularly vulnerable to neurocognitive decline. Clinicians need to be prepared to address and treat all aspects of these patients' care.

## Data Availability

The corresponding author will be responsible to provide more relevant data upon request.

## Supplementary Materials

Supplementary Materials Daily doses of psychotropic medications in the first three months after admission; results of EEG, laboratory tests, and image studies.

## References

[1] López-Muñoz F., Alamo C., Cuenca E., Shen W. W., Clervoy P., Rubio G. (2005). History of the discovery and clinical introduction of chlorpromazine. *Annals of Clinical Psychiatry*.

[2] Divakar A., Kanchan S., Jha G., Mishra S., Sharma D., Rath S. K. (2021). Alcohol induced impairment/abnormalities in brain: role of microRNAs. *Neurotoxicology*.

[3] Perry C. J. (2016). Cognitive decline and recovery in alcohol abuse. *Journal of Molecular Neuroscience*.

[4] Knorr U., Simonsen A. H., Jensen C. S. (2022). Alzheimer's disease related biomarkers in bipolar disorder - a longitudinal one-year case-control study. *Journal of Affective Disorders*.

[5] Attaluri P., Castillo A., Edriss H., Nugent K. (2018). Thiamine deficiency: an important consideration in critically ill patients. *The American Journal of the Medical Sciences*.

[6] Westermeyer J. J., Soukup B., Mayer J., Lee K. (2021). Identifying, assessing, and treating Korsakoff syndrome patients: updated perspectives. *The Journal of Nervous and Mental Disease*.

[7] Ahmed S., Akadiri T. V., Ata S., Ayub S. (2021). An unusual presentation of catatonia in non-alcoholic Wernicke encephalopathy. *Cureus*.

[8] Ott M., Werneke U. (2020). Wernicke’s encephalopathy from basic science to clinical practice. Part 1: understanding the role of thiamine. *Therapeutic Advances in Psychopharmacology*.

[9] Victor M. (1990). MR in the diagnosis of Wernicke-Korsakoff syndrome. *AJR. American Journal of Roentgenology*.

[10] Oudman E., Wijnia J. W., Oey M. J., van Dam M. J., Postma A. (2021). Wernicke encephalopathy in schizophrenia: a systematic review. *International Journal of Psychiatry in Clinical Practice*.

[11] Popa I., Rădulescu I., Drăgoi A. M., Trifu S., Cristea M. (2021). Korsakoff syndrome: an overlook (review). *Experimental and Therapeutic Medicine*.

[12] Thomson A. D., Guerrini I., Marshall E. J. (2012). The evolution and treatment of Korsakoff's syndrome: out of sight, out of mind?. *Neuropsychology Review*.

[13] Jung Y. C., Chanraud S., Sullivan E. (2012). Neuroimaging of Wernicke's encephalopathy and Korsakoff's syndrome. *Neuropsychology Review*.

[14] Harper C. (1983). The incidence of Wernicke's encephalopathy in Australia--a neuropathological study of 131 cases. *Journal of Neurology, Neurosurgery, and Psychiatry*.

[15] Barata P. C., Serrano R., Afonso H., Luís A., Maia T. (2020). Wernicke-Korsakoff syndrome. *Prim Care Companion CNS Disord.*.

[16] Ikeda T., Sakurai K., Matsukawa N., Yoshida M. (2020). Atrophic mammillary bodies with hypointensities on susceptibility-weighted images: a case-study in Korsakoff syndrome. *Journal of the Neurological Sciences*.

[17] Vatsalya V., Li F., Frimodig J. (2021). Repurposing treatment of Wernicke–Korsakoff syndrome for Th-17 cell immune storm syndrome and neurological symptoms in COVID-19: thiamine efficacy and safety, in-vitro evidence and pharmacokinetic profile. *Frontiers in Pharmacology*.

[18] Bagash H., Marwat A., Marwat A., Kraus B. (2021). A case of chronic Wernicke encephalopathy (WE): an underdiagnosed phenomena. *Cureus.*.

[19] Reed L. J., Lasserson D., Marsden P. (2003). FDG-PET findings in the Wernicke-Korsakoff syndrome. *Cortex*.

