# Supplementary material for: Clinical evaluation and management of a 45-year-old man with confusion, psychosis, agitation, stereotyped behavior, and impaired speech
Source: Case Rep Psychiatry. 2022 May 17;2022:8162871. doi: 10.1155/2022/8162871 (PMC9130019; doi:10.1155/2022/8162871)
Supplement: Supplementary Materials — Daily doses of psychotropic medications in the first three months after admission; results of EEG, laboratory tests, and image studies. [file 8162871.f1.docx]

**Supplementary Appendix**

|  |
| --- |

Fig. 1: Daily doses of psychotropic medications in the first three months after admission. Each bar represents the average daily dose over a three-day period. Hydroxyzine was used as needed to treat agitation and anxiety. Clonazepam and lorazepam were administered as part of a scheduled regimen and on an as needed basis. Clonazepam 2 mg daily was administered in the first nineteen days and then switched to lorazepam. As-needed lorazepam was mostly used to treat agitation. Clozapine, valproic acid and galantamine were scheduled medications.

|  |  |  |
| --- | --- | --- |
| Blood lab test |  |  |
| Component |  | **Ref range** |
| WBC | 8.8 | 4.5 - 11.0 K/mcL |
| RBC | 4.17 | 4.00 - 5.70 M/mcL |
| Hemoglobin | 13.0 | 12.6 - 17.4 g/dL |
| Hematocrit | 39.4 | 37.0 - 50.0 % |
| MCV | 94.5 | 80.0 - 96.0 fL |
| MCH | 31.2 | 28.0 - 33.0 pg |
| MCHC | 33.0 | 33.0 - 36.0 g/dL |
| Platelets | 285 | 153 - 367 K/mcL |
| MPV | 10.7 | 9.4 - 12.4 fL |
| RDW | 13.2 | 12.0 - 15.2 % |
| Nucleated RBC % | 0.0 | % |
| Nucleated RBC Abs | 0.0 | K/mcL |
| WBC | 8.8 | 4.5 - 11.0 K/mcL |
|  |  |  |
| Sodium | 140 | 136 - 145 mmol/L |
| Potassium | 3.9 | 3.5 - 5.1 mmol/L |
| Chloride | 102 | 98 - 107 mmol/L |
| CO2 | 31 High | 21 - 30 mmol/L |
| Glucose Bld | 79 | 70 - 99 mg/dL |
| Creatinine | 0.75 | 0.66 - 1.25 mg/dL |
| BUN | 19 | 9 - 20 mg/dL |
| Calcium | 9.4 | 8.6 - 10.2 mg/dL |
| Total Protein | 6.5 | 6.3 - 8.2 g/dL |
| Albumin | 3.7 | 3.5 - 5.2 g/dL |
| Bilirubin Total | <0.1 Low | 0.3 - 1.2 mg/dL |
| Alk Phos | 63 | 38 - 126 units/L |
| AST | 50 | 17 - 59 units/L |
| ALT | 26 | 0 - 49 units/L |
| Anion Gap | 8 | 4 - 16 |
| Hemoglobin A1C | 5.7 High | <=5.6 % |
| CK Total | 704 | 55 - 170 units/L |
|  |  |  |
| TSH | 0.69 | 0.47 - 4.68 mIU/L |
| T4 Free | 1.1 | 0.6 - 2.5 ng/dL |
|  |  |  |
| VITAMIN B1 | 308 | 70 - 180 nmol/L |
| Folate | 2.3 Low | >=4.0 ng/mL |
| Vitamin B 12 | 419 | 239 - 931 pg/mL |
| Vit D 25 Hydroxy | 36 | 20 - 80 ng/mL |
| Vitamin D2 25 Hydroxy | <4 | ng/ml |
| Vitamin D3 25 Hydroxy | 36 | Ng/ml |
| Magnesium | 2.0 | 1.6 - 2.6 mg/dL |
| Phosphorus | 3.6 | 2.5 - 4.5 mg/dL |
|  |  |  |
|  |  |  |
|  |  |  |
| HIV Ag/Ab | nonreactive | nonreactive |
| RPR | nonreactive | nonreactive |
| T Pallidum Ab (FTA-Ab) | nonreactive | nonreactive |
|  |  |  |
| Lipid profile |  |  |
| Cholesterol | 166 mg/dL | < 200 mg/dL |
| Triglycerides | 139 mg/dL | < 150 mg/dL |
| HDL | 1. mg/dL | >60 mg/dL |
| LDL Calc | 104 mg/dL | < 100 mg/dL |
|  |  |  |
| Carbamazepine Lvl | 8.0 mcg/mL | 4.0 - 12.0 mcg/mL |
|  |  |  |
| Hepatitis Panel |  |  |
| HBV Surface Ag | negative | negative |
| HCV Ab Interp | negative | negative |
| HAV IgM | negative | negative |
| HBV Core IgM | negative | negative |
|  |  |  |
| Sed Rate | 39 | 0 - 15 mm/hour |
| CRP | 6.3 | <=1.0 mg/dL |
| Thyroid Peroxidase Ab | 3.9 IU/mL | 0.0 - 9.0 IU/mL |
| ANA, HEp2, IgG | 1:80 | < 1:80 |
| dsDNA Antibody | 3 | 0-24 |
|  |  |  |
|  |  |  |
| Paraneoplastic Syd AB |  |  |
| CV2.1 Antibody IgG | <1:10 | <1:10 |
| PARANEO AB PNL | Non detected | Non detected |
| AMPHIPHYSIN AB, S | negative | negative |
| SOX1 Ab IgG | negative | negative |
|  |  |  |
| Sjogren'S Ab |  |  |
| SSA 52 (Ro) (ENA), IgG | 2 | 0 - 40 AU/mL |
| SSA 60 (Ro) (ENA), IgG | 2 | 0 - 40 AU/mL |
| SSB (La) (ENA), IgG | 0 | 0 - 40 AU/mL |
|  |  |  |
| CSF |  |  |
| Protein CSF | 19 | 12 - 60 mg/dL |
| Glucose CSF | 71 | 40 - 70 mg/dL |
| WBC CSF | 0 | 0 - 5 /mcL |
| RBC CSF | 3 High | 0 - 0 /mcL |
| Polys % CSF | 0 | % |
| Lymphs % CSF | 100 | % |
| CSF PRE-CENTRIFUGE COLOR | Colorless |  |
| CSF PRE-CENTRIFUGE CLARITY | Clear |  |
| CSF POST CENTRIFUGE COLOR | N/A |  |
| CSF POST CENTRIFUGE CLARITY | N/A |  |
| NMDA receptor Ab CSF | < 1:1 | <1:1 |
| VDRL CSF | non reactive | non reactive |
| 14-3-3 Tau, Total, CSF | prion disease: <0.2% |  |
| RT-QuIC, CSF | Negative | negative |
| T-tau protein (CSF) | 127 pg/ml | 0-1149 pg/ml |
| 14-3-3 protein (CSF) | negative | negative |
| CSF Culture w Gram Stain | negative | negative |
| LYME DNA PCR | Not detected |  |
|  |  |  |

**Procedure:** **EEG:**

**Technique:** Procedure done was a 21-electrode video and digital Recording. Recording was routine. Recording measures by the international 10/20 system with electrodes applied with paste and impedances below 5000 ohms.  This study was performed in our laboratory. Other electrodes used were EKG. Conditions of the recording were: Awake. Activation procedures were: IPS; voice.

**Background:** Background activity: The awake background rhythm was characterized by continuous predominantly alpha frequency waveforms at normal voltages. There was a well-formed, symmetric, and reactive 9 Hz posterior dominant rhythm. Symmetry & focal abnormalities: The background was symmetric with no focal abnormalities. Sleep rhythms: Awake only, no N1 or N2 sleep transients. Epileptiform activity: No epileptiform discharges were seen. Seizures: No seizures were seen. Video events: No clinical events.

**Activation:** Intermittent photic stimulation (IPS) produced no EEG abnormalities. Hyperventilation was not performed due to COVID-19 restrictions. Other finding: EKG: Regular.

**Interpretation:** This is a normal EEG. No epileptiform discharges are seen. Clinical correlation: A normal EEG does not rule out the diagnosis of epilepsy. Clinical correlation is recommended.

**Image studies:**

**MRI of the brain and MR angiogram of the intracranial arteries.**

TECHNIQUE:

Multiplanar, multi parametric imaging was performed without and with intravenous contrast

6.2 mL of Gadavist was administered intravenously. Maximum intensity projections were generated.

INDICATION: Acute change in mental status. Psychosis.

FINDINGS:

No evidence of acute intracranial hemorrhage or infarction.

No evidence of hydrocephalus

Moderate nonspecific confluent T2 hyperintensity in the periventricular white matter of both cerebral hemispheres

No abnormal enhancement in the brain parenchyma or leptomeninges

Intracranial arteries are patent. No evidence of aneurysms.

No abnormal enhancement in vessel walls

IMPRESSION: No acute intracranial abnormalities. No evidence of vessel wall enhancement of the intracranial arteries.

**PET Brain/Head Imaging Metabolic Eval**

CLINICAL: 45 y.o. M with Bipolar disorder, psychosis, memory loss, depression, and anxiety presenting for concerns of frontal/temporal dementia.

The patient is non-diabetic and weighs 140 lbs, with fasting blood glucose prior to FDG injection of 80 mg/dl.

PROCEDURE: Following our standard protocol, the patient fasted for a minimum of 4 hours prior to the intravenous injection of 8.08 mCi F-18-FDG. Imaging began 93 minutes following injection. The images were obtained from the top skull to the neck, reconstructed in the sagittal, transverse and coronal axes, and viewed using volume rendering. Standardized uptake values (SUV) were computed for abnormal regions. A localizing CT scan was performed to image the head/neck for attenuation purposes. The CT images were not obtained for the diagnostic purposes.

COMPARISON: MRI brain 9/13/2021.

COMMENT: FDG PET/CT images of the brain demonstrate normal radiotracer distribution in the bilateral frontal, parietal, and most temporal lobes (SUV = 8.6-9.58). There is relative decrease in metabolic activity in the bilateral occipital (SUV = 4.6 in the right; SUV = 4.2 in the left), left inferior area of temporal lobe (SUV = 5.5), and cerebellum bilaterally (SUV = 5-6), without abnormal lesions on CT.

IMPRESSION: FDG PET/CT imaging of the brain shows relative decrease in metabolic activity in bilateral occipital, left inferior area of temporal lobe, and cerebellum bilaterally compared to the normal radiotracer distribution in the bilateral cerebral hemispheres. These findings are non-specific and clinic correlation is recommended.
